# Overview of (1→3)-β-D-glucan immunobiology

**DOI:** 10.1080/09629359791550

**Published:** 1997-11

**Authors:** David L. Williams

**Affiliations:** 1 Department of Surgery James H. Quillen College of Medicine Box 70575 Johnson City TN 37614-0002 USA

## Abstract

Glucans are (1→3)-β-D-glucose polymers that are found in the cell wall of fungi, bacteria and plants. Glucans are known to stim ulate humoral and cell-mediated immunity in humans and animals. In addition to the potent immune stimulatory effects of (1→3)-β-D-glucans, there are a number of toxicological effects associated with exposure to the water-insoluble, microparticulate form of the polymer. Recent investigations have suggested a potential role for (1→3)-β-D-glucans in inhalational toxicity. Specifically, (1→3)-β-D-glucans have been implicated in the symptomatology associated with ‘sick building’ syndrome. The mechanisms by which (1→3)-β-D-glucans mediate immune stimulation and, perhaps, toxicity are currently under investigation. It is now established that (1→3)-β-D-glucans are recognized by macrophages and, perhaps, neutrophils and natural killer cells via a (1→3)-β-glucan specific receptor. Following receptor binding, glucan modulates macrophage cytokine expression. Here we review the chemistry, immunobiology and toxicity of (1→3)-β-D-glucans and how it may relate to effects caused by inhalation.


Mediators of Inflammation, **6,** 247–250 (1997)

### Overview of (1→3)-β-D-glucan immunobiology

**David L. Williams**

Department of Surgery, James H. Quillen College of Medicine, Box 70575, Johnson City, TN 37614-0002, USA

**Glucans are (1→3)-β-D-glucose polymers that are found in the cell wall of fungi, bacteria and plants. Glucans are known to stimulate humoral and cell-mediated immunity in humans and animals. In addition to the potent immune stimulatory effects of (1→3)-β-D-glucans, there are a number of toxicological effects associated with exposure to the water-insoluble, microparticulate form of the polymer. Recent investigations have suggested a potential role for (1→3)-β-D-glucans in inhalational toxicity. Specifically, (1→3)-β-D-glucans have been implicated in the symptomatology associated with 'sick building' syndrome. The mechanisms by which (1→3)-β-D-glucans mediate immune stimulation and, perhaps, toxicity are currently under investigation. It is now established that (1→3)-β-D-glucans are recognized by macrophages and, perhaps, neutrophils and natural killer cells via a (1→3)-β-D-glucan specific receptor. Following receptor binding, glucan modulates macrophage cytokine expression. Here we review the chemistry, immunobiology and toxicity of (1→3)-β-D-glucans and how it may relate to effects caused by inhalation.**

**Keywords:** Glucan polymers, glucan chemistry, immunobiology, glucan toxicity

### Introduction

Glucans are polymers of glucose that are widely distributed throughout the biosphere [1]. Specifically, glucans are found in the cell walls of plants, bacteria and fungi, as minor constituents of fungal cytosol and as polymers which are secreted into the environment by glucan-producing microorganisms [1]. Glucans can be broadly classified according to the type of intrachain linkage of the polymer, as α- or β-linked [1]. The β-linked glucans are the predominant form found in fungi [1]. It is the fungal-derived (1→3)-β-D-glucans which have been reported to modulate various aspects of immunity [2–5]. In the fungal cell wall, (1→3)-β-D-glucans are linked to proteins, lipids and other carbohydrates such as mannan [1].

The specific function of glucans in the physiology of fungi is not clearly understood. However, it is generally considered that the primary function of these polymers is a structural one in that they help to maintain the rigidity and integrity of the fungal cell wall [1]. The glucan polymers in the fungal cell wall may form a meshwork, due to the presence of (1→6)-β-D-glucopyranose side-chain branches, which may connect adjacent (1→3)-β-D-glucan polymers [1]. Fig. 1 shows the basic structure for a non-branched (1→3)-β-D-glucan polymer and for a (1→3)-β-D-glucan polymer with single (1→6)-β side-chain branches.

Glucan polymers can exist as a single polymer strand with a helical conformation (single helix) or as a stable complex of three polymer strands forming a triple helix [6]. The triple helix, which appears to be the preferred form, is stabilized by extensive hydrogen bonding at the O-2 hydroxyl [7–10].

### Immunologic effects of (1→3)-β-D-glucans

The ability of naturally occurring complex polysaccharide polymers to modulate immunity has been well documented [2–5]. In 1959, Benacerraf *et al.* [11] demonstrated that zymosan, a glucomannan isolated from *Saccharomyces cerevisiae*, produced marked hyperplasia and hyperfunctionality in fixed tissue macrophages. Di Luzio and Morrow [12], Cutler [13] and Kelly and colleagues [14] confirmed and extended the work of Benacerraf *et al.* [11]. In 1961, Riggi and Di Luzio [15] demonstrated that glucan, a (1→3)-β-D-linked glucopyranose polymer, was the macrophage-stimulating agent in zymosan. Numerous studies have shown that (1→3)-β-D-glucan polymers will increase the function of macrophages [16–19], neutrophils [20,21] and other immunocytes. These observations have stimulated investigation into the potential biomedical applications of polymeric (1→3)-β-D-glucans [4]. Unfortunately, there were also toxicological effects associated with the systemic admin-

© 1997 Rapid Science Publishers

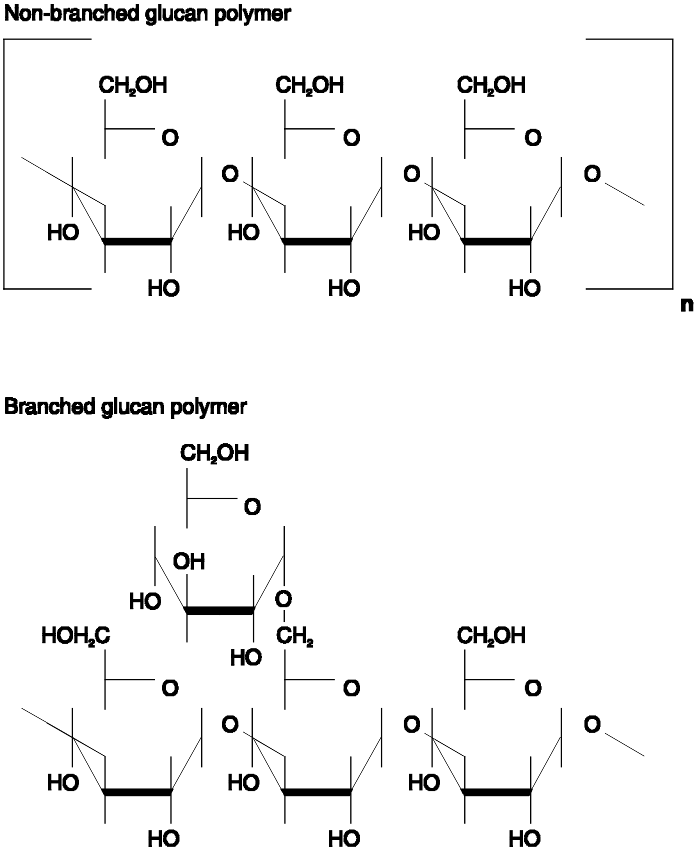

FIG. 1. Primary structure of a single, non-branched and a single, branched (1→3)-β-D-glucan polymer. The backbone of the glucan polymer is composed of glucose subunits connected by intrachain glycosidic (1→3)-β-linkages. In a branched (1→3)-β-D-glucan polymer, the branches are connected by (1→6)-β-linkages. In this model, the side-chain branches are a single glucose subunit. Glucan polymers can exist as a single polymer strand with a helical conformation (single helix) or as a stable complex of three polymer strands forming a triple helix. The triple helical form is generally considered to be the preferred form in nature.

istration of these agents. Upon initial isolation from yeast, (1→3)-β-D-glucans are usually water-insoluble microparticulates [6]. Systemic (intravenous) administration of glucan microparticulates is associated with hypertrophy and hyperplasia of macrophage-rich organs such as the liver, lung and spleen. Specifically, granuloma formation is observed [22]. Interestingly, many workers have reported that if (1→3)-β-D-glucans are converted to a water-soluble form, the immunologic activity is preserved, but the undesirable side effects are eliminated [23].

The studies by Rylander and Fogelmark and colleagues [24–28] suggest that the effects of (1→3)-β-D-glucans in pulmonary symptomatology may include synergy with endotoxin. Therefore, an important aspect of glucan's immunobiological activity is its adjuvant effect. Numerous studies have demonstrated the ability of (1→3)-β-D-glucans to exert adjuvanticity when combined with a variety of bacterial, viral or parasite vaccines [29–36]. Glucan has been shown to increase humoral and cell-mediated immunity to antigens [29–36]. In addition, glucans have demonstrated additive or synergistic effects on immunity when combined with a variety of agents [17,19].

### Receptor-mediated binding of a (1→3)-β-D-glucan to the human macrophage cell line U937

The cellular and molecular mechanisms by which (1→3)-β-D-glucans modulate immunity are just beginning to emerge. We have shown that glucans mediate immunological activity, in part, via macrophage participation [17,19]. Fogelmark *et al.* [28] have speculated that glucans mediate pulmonary effects due, in part, to their effect on macrophages. The first step in the interaction of glucan with mammalian macrophages is thought to involve the binding of (1→3)-β-D-glucan to a macrophage receptor [37–40]. The *in vivo* significance of these receptor studies is uncertain, because virtually all of these experiments were limited to *in vitro* phagocytosis and/or phagocytosis inhibition assays [21,37,38]. More importantly, virtually all of these studies used water-insoluble glucans which were not chemically characterized [21,37, 38].

To conclusively demonstrate the presence of a (1→3)-β-D-glucan-specific receptor requires a water-soluble, chemically characterized, radiolabeled glucan ligand. We have developed a process which achieves these goals [6]. Preliminary data with the human promonocytic cell line U937 indicate that glucan phosphate binding obeys the criterion for specific binding in that competition for the binding sites can be demonstrated in the presence of a 10-fold excess of unlabeled ligand [41].

Association binding studies were also undertaken. In these experiments, U937 ($1 \times 10^6$ cells) were co-incubated with varying concentrations of $^3$H-glucan phosphate for 90 min (Fig. 2).

We observed a rate constant ($K_{ob}$) of 0.95 $min^{-1}$, a dissociation constant ($K_D$) of 37 µmol/l and a maximum binding ($B_{max}$) of $6.5 \times 10^7$ sites/cell. This indicates a medium-affinity receptor and rapid binding of the ligand. The $B_{max}$ value more than accounted for the entire U937 cell surface area. This suggested that the glucan was being internalized following receptor binding.

Konopski *et al.* [42] have reported that murine macrophages will bind and internalize a fluorescent labeled glucan, which is consistent with our observations. We have examined U937 cells by electron microscopy following glucan exposure and observed an increase in phagolysosome formation which is consistent with uptake of glucan [41]. We conclude that the binding and internalization of glucans by human macrophages is a two-phase process; the first phase is a rapid binding of the glucan ligand to the receptor and is followed by a slower uptake/internalization phase. In subsequent studies, we examined the specificity of the (1→3)-β-D-glucan receptor on U937 cells. In this series of experiments, U937 ($1 \times 10^6$ cells) was incubated with $^3$H-glucan phosphate and varying concentrations of unlabeled glucan phosphate, pullulan, a (1→4)-α-linked glucose polymer and schizophyllan, a branched (1→3)-β-D-glucan polymer, for 90 min.

In this competitive binding study (Fig. 3), pullulan did not compete for binding to the (1→3)-β-D-glucan receptor. Schizophyllan showed a fivefold increase in affinity for the human macrophage (1→3)-β-D-glucan receptor compared to non-branched glucan phosphate. These data conclusively demonstrate the presence of a (1→3)-β-D-glucan-specific receptor on a human macrophage-like cell line. Further, these data demonstrate that the glucan receptor is specific for (1→3)-β-linked polymers and that the glucan receptor has a higher affinity for one form of glucan over another, that is, branched versus non-branched.

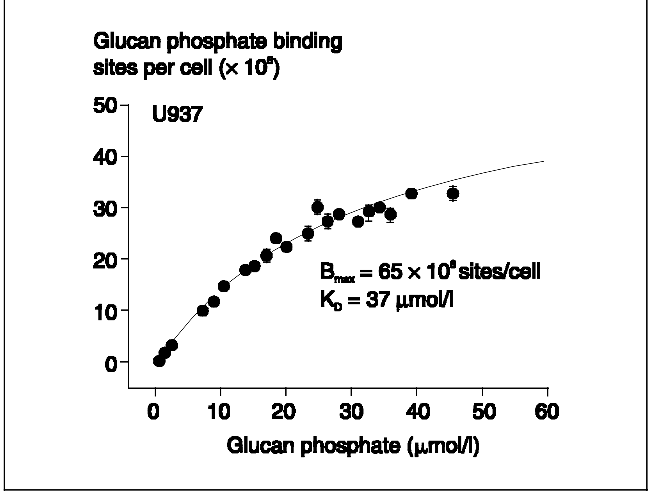

FIG. 2. Specific binding and internalization of glucan phosphate to U937 cells. Combined saturation and competitive displacement data yielded specific binding (sites per cell) to U937 as a function of (1→3)-β-D-glucan phosphate molarity at 37°C. We mathematically derived the dissociation constant ($K_D$) and maximum binding ($B_{max}$) value. The results are given as means and 95% confidence intervals of means ± SEM from at least four replicates and represent data from five separate experiments.

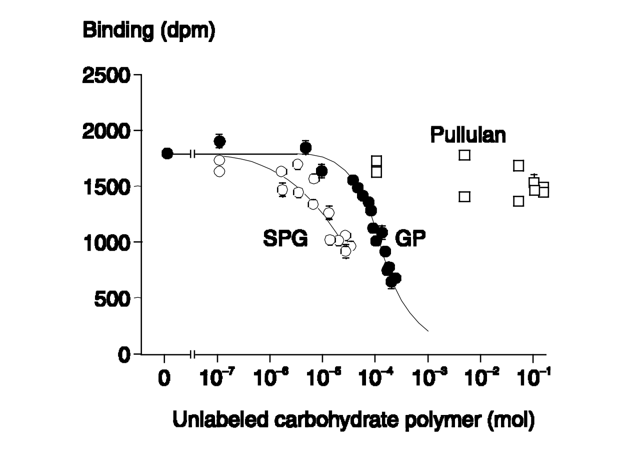

FIG. 3. Competitive displacement of tritiated glucan phosphate and schizophyllan at 37°C. Pullulan (open squares) does not compete for the same binding sites as unlabeled glucan phosphate (GP, closed circles). Schizophyllan (SPG, open circles) does compete for the same binding as unlabeled glucan phosphate and has an approximate fivefold increase in affinity for the binding site. The results are shown as means ± SEM of at least four replicates from two separate experiments.

### Conclusions

Indirect evidence continues to mount concerning the potential involvement of (1→3)-β-D-glucans in airways inflammation. The data thus far suggest that glucans must act in concert with other etiologic agents, such as endotoxin, in order to mediate symptomatology. Therefore, the primary role of (1→3)-β-D-glucans appears to be as an adjuvant which exerts an additive or synergistic effect when combined with other agents.

### References

1. Ruiz-Herrera J: Biosynthesis of beta-glucans in fungi. *Antonie Van Leeuwenhoek* 1991, **60**:72–81.
2. Williams D, Mueller A, Browder W: Prevention and clinical evaluation of carbohydrate immunopharmaceuticals in the prevention of sepsis and septic sequelae. *J Endotoxin Res* 1995, **2**:203–208.
3. Williams DL, Pretus HA, McNamee RB, *et al.*: Development, physicochemical characterization and preclinical efficacy evaluation of a water soluble glucan sulfate derived from *Saccharomyces cerevisiae*. *Immunopharmacology* 1991, **22**:139–156.
4. Browder W, Williams D, Pretus H, *et al.*: Beneficial effect of enhanced macrophage function in the trauma patient. *Ann Surg* 1990, **211**:605–613.
5. Williams D, Browder W: Development of natural product (1→3)-β-D-glucan polymers as immune stimulating pharmaceuticals. *Polymer Adv Tech* 1994, **5**:529–534.
6. Williams DL, McNamee RB, Jones EL, *et al.*: A method for the solubilization of a (1→3)-β-D-glucan isolated from *Saccharomyces cerevisiae*. *Carbohyd Res* 1991, **219**:203–213.
7. Ensley HE, Tobias B, Pretus HA, *et al.*: NMR spectral analysis of a water-insoluble (1→3)-β-D-glucan isolated from *Saccharomyces cerevisiae*. *Carbohyd Res* 1994, **258**:307–311.
8. Marchessault RH, Deslandes Y: Fine structure of (1→3)-D-glucans: curdlan and paramylon. *Carbohyd Res* 1994, **75**:231–242.
9. Deslandes Y, Marchessault RH, Sarko A: Triple-helical structure of (1→3)-β-D-glucan. *Macromolecules* 1980, **13**:1466–1471.
10. Bluhm TL, Deslandes Y, Marchessault RH, Perez S, Rinaudo M: Solid-state and solution conformation of scleroglucan. *Carbohyd Res* 1982, **100**:117–130.
11. Benacerraf B, Thorbecke GJ, Jacoby D: Effect of zymosan on endotoxin toxicity in mice. *Proc Soc Exp Biol Med* 1959, **100**:796–799.
12. Di Luzio NR, Morrow SH: The fate of foreign red cells in mice with altered reticuloendothelial function. *Proc Soc Exp Biol Med* 1965, **119**:647–652.
13. Cutler JL: The enhancement of hemolysin production in the rat by zymosan. *J Immunol* 1960, **84**:416–419.
14. Kelly LS, Dobson EL, Finney CR, Hirsch JD: Proliferation of the reticuloendothelial system in the liver. *Am J Physiol* 1960, **198**:1134–1138.
15. Riggi SJ, Di Luzio NR: Identification of a reticuloendothelial stimulating agent in zymosan. *Am J Physiol* 1961, **200**:297–300.
16. Sherwood ER, Williams DL, McNamee RB, Jones EL, Browder IW, Di Luzio NR: *In vitro* tumoricidal activity of resting and glucan-activated Kupffer cells. *J Leukocyte Biol* 1987, **42**:69–75.
17. Browder W, Williams D, Sherwood E, McNamee R, Jones EL, Di Luzio NR: Synergistic effect of nonspecific immunostimulation and antibiotics in experimental peritonitis. *Surgery* 1987, **102**:206–214.
18. Williams DL, DiLuzio NR: Glucan-induced modification of murine viral hepatitis. *Science* 1980, **208**:67–69.
19. Williams DL, Sherwood ER, McNamee RB, Jones EL, Browder IW, Di Luzio NR: Chemoimmunotherapy of experimental hepatic metastases. *Hepatology* 1987, **7**:1296–1304.
20. Williams DL, Sherwood ER, Browder IW, McNamee RB, Jones EL: Effect of glucan on neutrophil dynamics and immune function in *Escherichia coli* peritonitis. *J Surg Res* 1988, **44**:54–61.
21. Williams JD, Topley N, Alobaidi HM, Harber MJ: Activation of human polymorphonuclear leucocytes by particulate zymosan is related to both its major carbohydrate components: glucan and mannan. *Immunology* 1986, **58**:117–124.
22. Williams DL, Sherwood ER, McNamee RB, Jones EL, Di Luzio NR: Therapeutic efficacy of glucan in a murine model of hepatic metastatic disease. *Hepatology* 1985, **5**:198–206.
23. Williams DL, Sherwood ER, Browder IW, McNamee RB, Jones EL: Preclinical safety evaluation of soluble glucan. *Int J Immunopharmacol* 1988, **10**:405–411.
24. Goto H, Yuasa K, Rylander R: (1→3)-β-D-glucan in indoor air, its measurement and *in vitro* activity. *Am J Ind Med* 1994, **25**:81–83.
25. Fogelmark B, Sjöstrand M, Rylander R: Pulmonary inflammation induced by repeated inhalations of (1→3)-β-D-glucan and endotoxin. *Int J Exp Pathol* 1994, **75**:85–90.
26. Rylander R, Fogelmark B: Inflammatory responses by inhalation of endotoxin and (1→3)-β-D-glucan. *Am J Ind Med* 1994, **25**:101–102.
27. Fogelmark B, Goto H, Yuasa K, Marchat B, Rylander R: Acute pulmonary toxicity of inhaled β-1,3-glucan and endotoxin. *Agents Actions* 1992, **35**:50–56.
28. Fogelmark B, Sjöstrand M, Rylander R: Pulmonary inflammation induced by repeated inhalations of β(1,3)-D-glucan and endotoxin. *Int J Exp Pathol* 1994, **75**:85–90.
29. Holbrook TW, Cook JA, Parker BW: Glucan-enhanced immunogenicity of killed erythrocytic stages of *Plasmodium berghei*. *Infect Immun* 1981, **32**:542–546.
30. Holbrook TW, Cook JA, Parker BW: Immunization against *Leishmania donovani*: glucan as an adjuvant with killed promastigotes. *Am J Trop Med Hyg* 1981, **30**:762–768.
31. Cook JA, Holbrook TW: Immunogenicity of soluble and particulate antigens from *Leishmania donovani*: effect of glucan as an adjuvant. *Infect Immun* 1983, **40**:1038–1043.
32. Holbrook TW, Cook JA: Non-specific and specific stimulation of resistance against *Leishmania donovani* in C57Bl/6J Mice. *Ann Clin Lab Sci* 1983, **13**:411–417.
33. Holbrook TW, Cook JA: Immunization of mice against *Leishmania donovani* by subcutaneous injections of dead promastigotes. *Am J Trop Med Hyg* 1983, **32**:51–53.
34. Benach JL, Habicht GS, Holbrook TW, Cook JA: Glucan as an adjuvant for a murine *Babesia microti* immunization trial. *Infect Immun* 1982, **35**:947–951.
35. Lasarow RM, Williams DL, Theis JH: Humoral response following immunization with *Leishmania infantum* (ex. Oklahoma): a comparison of adjuvant efficacy in the antibody responses of Balb-C mice. *Int J Immunopharmacol* 1992, **14**:767–772.
36. Williams DL, Yaeger RG, Pretus HA, Browder IW, McNamee RB, Jones EL: Immunization against *Trypanosoma cruzi*: adjuvant effect of glucan. *Int J Immunopharmacol* 1989, **11**:403–410.
37. Abel G, Czop JK: Stimulation of human monocyte β-glucan receptors by glucan particles induces production of TNFα and IL-β. *Immunopharmacol* 1992, **14**:1363–1373.
38. Janusz MJ, Austen KF, Czop JK: Isolation of a unit ligand for β-glucan receptors of human monocytes [abstract]. *Int J Immunopharmacol* 1988, **10**:89.
39. Janusz MJ, Austen KF, Czop JK: Isolation of a yeast heptaglucoside for monocyte phagocytic β-glucan receptors [abstract]. *FASEB J* 1989, **3**:6421.
40. Czop JK, Kay J: Isolation and characterization of β-glucan receptors on human mononuclear phagocytes. *J Exp Med* 1991, **173**:1511–1520.
41. Muller A, Rice PJ, Ensley HE, *et al.*: Receptor binding and internalization of a water-soluble (1→3)-β-D-glucan biologic response modifier in two monocyte/macrophage cell lines. *J Immunol* 1996, **156**:3418–3425.
42. Konopski Z, Fandrem J, Selejid R, Eskeland T: Interferon inhibits endocytosis of soluble animated β-1,3-D-glucan and neutral red in mouse peritoneal macrophages. *J Interferon Cytokine Res* 1995, **15**:597–603.
